# PD166 Artificial Intelligence, Healthcare System Budget Cuts, And Flow of New Evidence: Moving To Living Health Technology Assessment Reform

**DOI:** 10.1017/S0266462324003921

**Published:** 2025-01-07

**Authors:** Grammati Sarri, Seye Abogunrin

## Abstract

**Introduction:**

Health technology assessment (HTA) agencies struggle with how to ensure timely assessment of promising technologies, especially considering the volume of rapidly produced evidence using complex analytical methodologies and applications, such as artificial intelligence (AI). Furthermore, healthcare systems that are already overburdened are now dealing with issues related to sustainability and increasing budgetary constraints resulting from several public health emergencies, such as the COVID-19 pandemic.

**Methods:**

A targeted literature review of primary publications in English published during the last five years was conducted to answer the following research question: Would AI integration into health outcomes research and health economics encourage automation in the HTA process, allowing for a living model—a real-time, dynamic approach using explicit methods to determine the value of a technology at different points in its lifecycle—to be implemented? We selected publications presenting information on the following concepts: automation in evidence generation; health economics in the decision-making context; cost efficiencies from the integration of automation; and separation of concepts such as lifecycle and living HTA. A narrative synthesis was conducted.

**Results:**

The publications selected explored four different aspects of the living concept in decision-making: living clinical guidelines, living evidence reviews and economic evaluations, and living HTA. Automation in systematic reviews (screening and data extraction), including time efficiencies, was the most frequently reported living aspect. The value of open-source economic models was increasingly recognized. Few references were found for methods such as living meta-analyses or network meta-analyses. Adaptive HTA was another related key term. A few publications outlined how a living HTA model could be implemented in real decision-making and its operational challenges.

**Conclusions:**

So far, HTA bodies have been slow in adopting AI and automation innovation in their practices. Pressures to evolve with the increasingly complex treatment and evidence landscape necessitate a reform in HTA methods. A living HTA model may overcome these barriers and ensure faster patient access for new, promising technologies. A set of “living” standards is needed to gain HTA trust.

